# The Role of Psychological Variables in Predicting Rehabilitation Outcomes After Spinal Cord Injury: An Artificial Neural Networks Study

**DOI:** 10.3390/jcm13237114

**Published:** 2024-11-25

**Authors:** Marta Mascanzoni, Alessia Luciani, Federica Tamburella, Marco Iosa, Emanuela Lena, Sergio Di Fonzo, Valerio Pisani, Maria Carmela Di Lucente, Vincenzo Caretti, Lucia Sideli, Gaia Cuzzocrea, Giorgio Scivoletto

**Affiliations:** 1 Spinal Center and Spinal Rehabilitation Laboratory, IRCCS Fondazione Santa Lucia, 00179 Rome, Italy; m.mascanzoni@lumsa.it (M.M.); luciani.1754443@studenti.uniroma1.it (A.L.); e.lena@hsantalucia.it (E.L.); s.difonzo@hsantalucia.it (S.D.F.); v.pisani@hsantalucia.it (V.P.); mc.dilucente@hsantalucia.it (M.C.D.L.); g.scivoletto@hsantalucia.it (G.S.); 2 Department of Human Sciences, LUMSA University of Rome, 00193 Rome, Italy; v.caretti@lumsa.it (V.C.); l.sideli@lumsa.it (L.S.); g.cuzzocrea1@lumsa.it (G.C.); 3 Department of Life Sciences, Health and Health Professions, Link Campus University of Rome, 00165 Roma, Italy; 4 Department of Psychology, Sapienza University of Rome, 00185 Roma, Italy; marco.iosa@uniroma1.it; 5 Smart Lab, IRCCS Fondazione Santa Lucia, 00179 Rome, Italy

**Keywords:** spinal cord injury, functional outcome, psychological variables, artificial neural networks

## Abstract

**Background**: Accurate prediction of neurorehabilitation outcomes following Spinal Cord Injury (SCI) is crucial for optimizing healthcare resource allocation and improving rehabilitation strategies. Artificial Neural Networks (ANNs) may identify complex prognostic factors in patients with SCI. However, the influence of psychological variables on rehabilitation outcomes remains underexplored despite their potential impact on recovery success. **Methods:** A cohort of 303 patients with SCI was analyzed with an ANN model that employed 17 input variables, structured into two hidden layers and a single output node. Clinical and psychological data were integrated to predict functional outcomes, which were measured by the Spinal Cord Independence Measure (SCIM) at discharge. Paired Wilcoxon tests were used to evaluate pre–post differences and linear regression was used to assess correlations, with Pearson’s coefficient and the Root Mean Square Error calculated. **Results**: Significant improvements in SCIM scores were observed (21.8 ± 15.8 at admission vs. 57.4 ± 22.5 at discharge, *p* < 0.001). The model assigned the highest predictive weight to SCIM at admission (10.3%), while psychological factors accounted for 36.3%, increasing to 40.9% in traumatic SCI cases. Anxiety and depression were the most influential psychological predictors. The correlation between the predicted and actual SCIM scores was R = 0.794 for the entire sample and R = 0.940 for traumatic cases. **Conclusions**: The ANN model demonstrated the strong impact, especially for traumatic SCI, of psychological factors on functional outcomes. Anxiety and depression emerged as dominant negative predictors. Conversely, self-esteem and emotional regulation functioned as protective factors increasing functional outcomes. These findings support the integration of psychological assessments into predictive models to enhance accuracy in SCI rehabilitation outcomes.

## 1. Introduction

Spinal cord injury (SCI) is a severe and life-changing event, often resulting in psychopathological outcomes and disability, even in the long term [1]. Additionally, SCI significantly impacts social and professional domains [2].

Despite significant scientific progress in the knowledge and understanding of the pathogenesis and treatment, it remains a devastating condition involving motor, sensory, and autonomic dysfunctions below the injury level, which can be temporary or permanent [3]. Currently, there is no effective treatment to completely repair spinal cord neural damage and the management of this injury is mainly focused on supportive measures aimed at preventing additional complications and rehabilitation [4,5].

SCI remains a major global health concern, with an estimated 0.91 million new cases, 20.64 million prevalent cases, and 6.20 million years lived with disability worldwide [6]. Furthermore, SCI has countless expenses affecting healthcare systems, communities, and society, especially with invaluable lifetime healthcare costs for patients and their families according to clinical and socio-demographic characteristics [6,7]. Men are commonly more affected, accounting for 78% of new cases, while the onset age has shifted from approximately 20 to 45 years [8]. Traumatic causes of SCI, such as motor vehicle accidents, falls, and violence, account for the majority of cases globally, with motor vehicle accidents being the leading cause in younger populations and falls being more frequent among the elderly [9,10]. Non-traumatic SCI, which results from congenital or genetic disorders or acquired conditions, also accounts for a significant proportion of cases, despite the absence of epidemiological research. As healthcare systems improve and life expectancy increases, the prevalence of non-traumatic SCI is expected to rise, particularly in older adults [11].

The SCI condition represents a significant challenge in inpatient rehabilitation, requiring complex, multifaceted treatment approaches [12]. The main goal of the rehabilitation process following SCI is to restore functional independence, but the outcomes are highly variable and depend on numerous factors. The outcomes of SCI rehabilitation are traditionally prognosticated based on clinical and demographic variables, such as the lesion level and completeness of injury, age and sex, functional and clinical scale scores, complications, and comorbidities [13]. However, patients after SCI are at risk of experiencing both physical and psychological issues [14]. Individuals with SCI often experience significant stress, anxiety, and depression. In fact, patients with SCI tend to have higher levels of depression and stress as well as lower self-esteem compared to healthy individuals [2]. Previous studies indicate that patients with SCI often face acute psychological distress, which can exacerbate emotional and cognitive responses, further affecting rehabilitation outcomes [15]. A significant number of individuals with SCI may face mental health challenges following their injury, with mental health disorder diagnoses ranging from 10% to as much as one-third of affected individuals [16]. Several studies suggest that psychological variables, such as anxiety and depression, may significantly influence rehabilitation outcomes and be crucial to recovery success [17,18] and that they are also related to lower resilience [19]. Additionally, individuals with severe medical conditions, including those with SCI, often show reduced emotional expressiveness and traits associated with alexithymia, which is characterized by difficulty in recognizing and describing emotions and distinguishing between emotions and their physical sensations. It also involves challenges in experiencing and expressing emotional states [20]. This emotional dysregulation may adversely affect motivation for self-regulation and rehabilitation in the context of illness or disability [21,22,23,24]. In the literature, the correlation between alexithymia and SCI remains complex and not linear [25,26]. Conversely, protective psychological traits such as social skills, resilience, and high self-esteem are linked to better adaptation and improved recovery outcomes [27]. Despite this, the role of psychological variables in predictive models for SCI outcomes remains quite underexplored, creating a gap in our understanding and management of these patients in the context of SCI inpatient rehabilitation.

Moreover, both traumatic and non-traumatic spinal cord injuries achieved significant improvements across all rehabilitation domains with a standardized rehabilitation program, including a psychological one, supporting its overall efficacy [28]. Mental health outcomes, including depression, anxiety, and stress, were consistently observed across patients in the post-acute phase. However, individuals with non-traumatic spinal cord injuries are at higher risk for adverse mental health outcomes [29].

Rehabilitation outcomes often focus mainly on physical function, disregarding the impact of psychological variables, which is essential for assessing the need for psychological screening measures and improving the identification of rehabilitation needs [18]. While the literature regarding the impact of positive psychological variables on outcomes after SCI is limited, positive psychological factors significantly influence post-rehabilitation subjective well-being in individuals with SCI and are related to increased life satisfaction following SCI. This may provide a potential approach for interventions aimed at facilitating positive outcomes [30].

Artificial Neural Networks (ANNs) have recently emerged as a more accurate method for differentiating classes of potential prognostic factors and predicting outcomes in neurorehabilitation patients, including those with spinal cord injuries. ANNs show significant potential in diagnosis, prognostication, management, rehabilitation planning, and the prevention of chronic complications [13,31,32].

Therefore, this study aims to provide a deeper understanding of the role of psychological variables in rehabilitation outcomes for patients with SCI to allow an improvement in rehabilitation strategies and clinical practice guidelines in the neurorehabilitation framework. We hypothesize that the percentage weight assigned to these psychological factors within the Artificial Neural Network (ANN) model significantly influences the prediction of SCIM scores at discharge. Additionally, we performed a secondary analysis on the subgroup of patients with traumatic injuries to investigate the role of psychological factors in these people for which spinal cord injury was a sudden and unexpected event.

## 2. Materials and Methods

### 2.1. Patients and Data

This study involved a secondary analysis of an extensive database containing demographic clinical and psychological data of patients with SCI admitted to the Spinal Centre of an Italian rehabilitation hospital (I.R.C.C.S. Fondazione Santa Lucia of Rome). The database comprises 1308 patients and has been previously utilized in several studies [13] and enriched with additional data. The focus of the present study was patients aged over 18 years admitted between 2017 and 2023 after their first SCI. The exclusion criteria were patients with cognitive impairments that compromised their ability to fully engage in the neurorehabilitation program or interfered with an accurate outcome assessment, individuals who experienced a discharge or transfer lasting more than three weeks before readmission (as these cases were classified as secondary admissions), patients with a shorter length of stay than seven days, and those who died during their rehabilitation period. As our hospital functions as a research institution, all the patients provided informed consent at the time of admission, authorizing the utilization of their clinical data for translational research purposes (General Data Protection Regulation, article 6.1 letter e, article 9.2 letter j and article 89. Italian Legislative Decree 30 June 2003, n. 196, Privacy Code, Article 110 bis).

### 2.2. Clinical Assessment and Neurorehabilitation

At admission, the database was populated with key variables: three continuous variables, including age, the Spinal Cord Independence Measure (SCIM) score (version II or III) [33,34], and the Walking Index for Spinal Cord Injury (WISCI) score; two ordinal variables, which included the American Spinal Injury Association (ASIA) Impairment Scale (AIS) score [35] (A: motor and sensory complete; B: motor complete, sensory incomplete; C: motor and sensory incomplete with low strength in the affected limbs; D: motor and sensory incomplete with a strength of 3 or more in the affected limbs) and the lesion level (cervical, thoracic, or lumbar); and several binary variables, such as gender, etiology (traumatic or non-traumatic), surgical intervention (yes/no), and the presence of complications like pressure sores(yes/no), heterotopic ossifications (HOs) (yes/no), respiratory complications (yes/no), pulmonary embolism (yes/no), deep vein thrombosis (yes/no), urologic complications (excluding urinary infections) (yes/no), preservation of motor tracts (motor complete/incomplete) (yes/no), and other complications (yes/no).

The psychological assessment battery utilized validated instruments to measure various constructs, as detailed below. The trait version of the State–Trait Anxiety Inventory (STAI-T), a 20-item self-report measure of trait anxiety, has been commonly utilized to examine ordinary levels of worrying and the general anxiety state in daily life in both general populations and individuals with SCI [28,36,37,38]. The Depression Questionnaire (DQ) of the Cognitive Behavioural Assessment 2.0 is a well-validated self-report measure of depression. It consists of 24 dichotomous items adapted from the Beck Depression Inventory and the Zung Self-Rating Depression Scale. It was used to evaluate clinical depression by asking individuals to identify their current experience of each symptom as well in patients with SCI [37,38]. The ‘cut-off’ score is set at one standard deviation above the normative mean, which is consistent with prior studies. Patients were classified as ‘anxious’ or ‘depressed’ if their scores on the respective scales exceeded the mean score of uninjured subjects by more than one standard deviation. The Eysenck Personality Questionnaire (EPQ) Extroversion scale (E Scale) consists of 12 dichotomous items, with each response scoring 0 or 1. A higher score indicates higher extroversion. This questionnaire enables the identification of personality traits that may develop into certain disorders or, conversely, constitute a protective factor against the possible onset of psychological problems [38,39]. The Rosenberg Self-Esteem Scale (RSES) is the standard measure for assessing global self-esteem. The scale comprises 10 items, 5 that are negatively worded, and employs a 4-point Likert response format, ranging from ‘strongly agree’ to ‘strongly disagree’. Higher scores indicate greater self-esteem, reflecting individuals with enhanced self-awareness and self-regard. Since self-esteem is considered a trait-like construct, the RSES may be particularly valuable as a moderator variable in various psychological studies [40]. The Toronto Alexithymia Scale-20 (TAS-20) is a 20-item Likert scale used to measure three-factor alexithymia—difficulties in identifying and describing feelings and externally oriented thinking. It focuses on the difficulties in regulating emotions instead of the effects and consequences of these issues [41]. Higher scores indicate higher alexythimia.

The SCIM score at discharge was examined as the main outcome of neurorehabilitation in this study.

### 2.3. ARtificial Intelligence Assistant for Neural Network Analysis (ARIANNA) and Statistical Analysis

Feed Forward Neural Network Multilayer Perceptron was utilized, comprising an input layer with seventeen variables, two hidden layers with five units each, and a single output node. The hyperbolic tangent function served as the activation function for all the units in both the hidden and output layers, and information flowed unidirectionally from input to output. Online training was used, with the following parameters: initial learning rate = 1.2, minimum learning rate = 0.001, training epochs = 10, momentum = 0.9, interval center = 0, interval offset = ±0.5, memory size = 1000, tolerance steps = 1, error change threshold = 0.0001, and error ratio = 0.001. Predictive scores were calculated to report the ANN results, including the raw percentage importance (RI) of each variable (summing to 100%) and the normalized importance (NI) relative to the most influential variable. All the data were used to train the ANN, without a separate testing split. This configuration, called ARtificial Intelligence Assistant for Neural Network Analysis (ARIANNA), was developed using the dedicated tool Neural Networks module of IBM Statistical Package for the Social Sciences software (SPSS, version 23) and was validated and used in previous studies on neurorehabilitation [42,43,44].

The continuous and ordinal variables were reported as the mean and standard deviation, while the binary and nominal variables were presented as percentage frequencies.

The pre–post comparisons were performed using the paired Wilcoxon test. The percentage weights assigned by the ANN to each variable were reported as a percentage of the total. The Pearson coefficient was used to assess the correlations, and linear fitting of the prediction versus actual outcomes was performed using the least squares method. The Mean Absolute Error (MAE) was used to assess the performance of the ANN.

The goodness of fitting was assessed using Pearson’s coefficient and the Root Mean Square Error (RMSE), which was:

$$RMSE=\frac{\sqrt{\sum_{n}^{1}\left({SCIM}_{i}\right)-Y_{i}}}{N}$$

The statistical significance threshold for all the analyses was set at 5%. All the statistical analyses, as well as the ANN, were performed using SPSS (Statistical Package for the Social Sciences) software of IBM, version 23.

## 3. Results

### 3.1. Cohort of All SCI Patients

The number of patients included in this analysis was 303, with a mean age of 52.3 ± 18.2 years. In total, 33% of them had a cervical lesion, 49% had a thoracic lesion, and 18% had a lumbar lesion. These and other demographic and clinical characteristics measured at admission into the rehabilitation hospital are reported in Table 1. The SCIM score at admission was 21.8 ± 15.8, and at discharge, it was 57.4 ± 22.5, with a statistically significant improvement (*p* < 0.001). The mean length of stay in the rehabilitation hospital was 166 ± 120 days.

**Figure 1:**
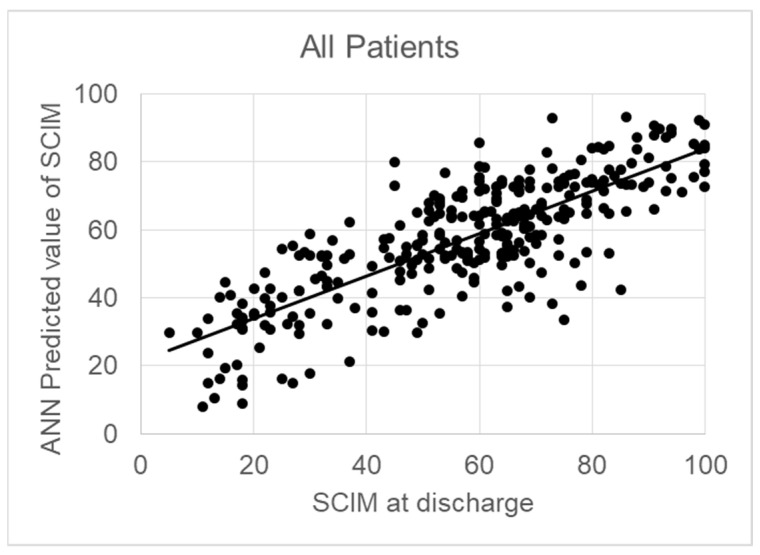
Correlation Between ANN-Predicted and Actual SCIM Scores at Discharge for the Whole Sample (R = 0.794, *p* < 0.001).

Table 1 also reports the percentage weight associated with each input variable for predicting the SCIM at discharge. The ASIA and SCIM scores at admission were the most important factors for predicting the SCIM score at discharge (weight: 10.7% and 10.3%, respectively). The sum of the weights associated with psychological factors (anxiety, depression, alexithymia, self-esteem, and extroversion) covered 36.3% of the total. The correlation between the predicted and actual SCIM score at discharge was *r* = 0.794 (*p* < 0.001). Figure 1 shows this regression, the formula of which was SCIM score at discharge = 0.62 × Predicted SCIM + 22, with an RMSE = 21.

### 3.2. Cohort of Patients with Traumatic Lesion

**Figure 2:**
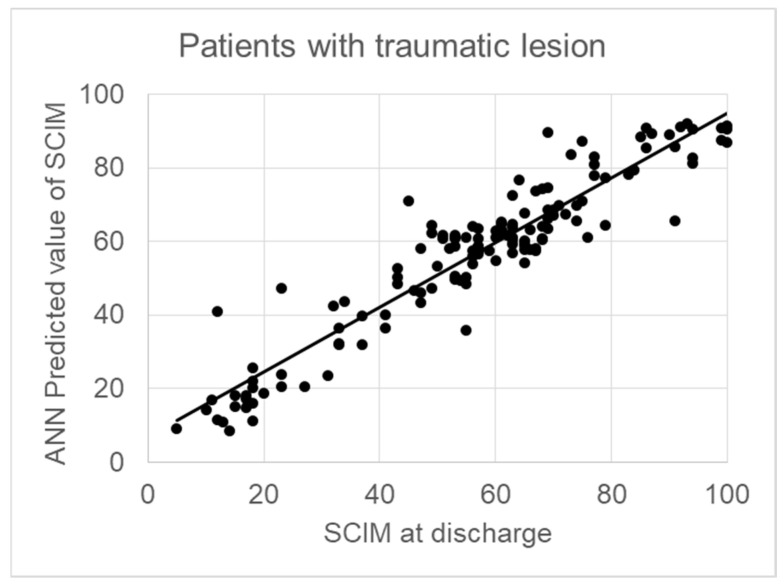
Correlation Between ANN-Predicted and Actual SCIM Scores at Discharge in Patients with Traumatic Lesions (R = 0.940, *p* < 0.001).

In 140 patients, the cause of the lesion was traumatic. The sum of the weights of the psychological factors increased to 40.9% in patients with traumatic lesions. Table 1 reports the demographical, clinical, and psychological variables in these patients, together with the weights assigned by the ANN to each one of these variables for predicting the SCIM at discharge. The correlation between the predicted and actual SCIM values at discharge was very high (*r* = 0.940, *p* < 0.001), with a regression line close to y = x (SCIM score at discharge = 0.88 × Predicted SCIM + 6.8) (Figure 2) and an RMSE = 5.

## 4. Discussion

This study aimed to assess the role of psychological factors in predicting rehabilitation outcomes following SCI using an ANN approach with data from a single Italian hospital. The potential of predicting the SCIM score at discharge from rehabilitation based on demographic, clinical, and psychological variables obtained at admission was evaluated in patients with both traumatic and non-traumatic SCI. The ARIANNA model, based on an ANN, allowed for the precise identification of the relative weight of each variable in predicting rehabilitation outcomes. The analysis indicated that the level of independence, as measured by the SCIM score at discharge, was primarily determined by clinical variables such as the SCIM score at admission, the lesion level (cervical, thoracic, lumbar), and the motor completeness of the injury (complete vs. incomplete injury). The ANN further highlighted the influence of age. These findings are consistent with previous studies that have highlighted that the SCIM baseline is a robust predictor of long-term functional outcomes [42] and the importance of lesion characteristics in determining the rehabilitation potential, such as the level and severity of SCI and age [13,45,46,47]. Psychological factors collectively accounted for 36.3% of the total predictive weight for functional outcomes, with even higher contributions (40.9%) observed in patients with traumatic injuries. Among the traumatic injury group, the most influential variables were anxiety and depression levels, with weights of 10.3% and 10.6%, respectively. These two variables contributed significantly to the model’s predictions. This aligns with previous research indicating that psychological health is integral to functional recovery in patients with SCI [18]. High levels of anxiety and depression have been associated with lower rehabilitation outcomes, as they can impede motivation and engagement in therapy [16,17]. Patients with SCI who exhibit depressive symptoms, including psychomotor disturbance, changes in appetite, and sleep disturbances, are likely to experience longer lengths of hospitalization. This extended hospitalization is associated with decreased mobility and functional independence at discharge compared to patients without these symptoms [47]. Moreover, depressive symptoms contribute to a slower rate of functional improvement and are linked to increased morbidity and mortality [46]. Anxiety can lead to extreme preventive behaviors, leading individuals to avoid situations they perceive as potentially anxiety-inducing or threatening. The prolonged persistence of such behaviors may result in disuse and increased disability, further diminishing rehabilitation opportunities [48]. On the contrary, protective psychological traits, such as resilience and high self-esteem, positively correlate with better adaptation and recovery outcomes, enabling individuals to effectively navigate the challenges associated with their injuries and encouraging active participation in rehabilitation for patients with SCI [27,49]. Similar results have been demonstrated in stroke and cardiological rehabilitation. In both cases, higher levels of depression at the onset of rehabilitation were significantly correlated with lower rehabilitation outcomes, specifically reflecting a decrease in exercise capacity and poorer emotional, physical, and social quality of life; conversely, anxiety levels showed a positive association with improvements in exercise capacity and emotional and physical quality of life [50,51]. However, also in stroke and cardiac rehabilitation, several psychological variables and emotional regulation significantly affect patient engagement and overall recovery outcomes. Specifically, increased self-efficacy is related to better functional outcomes, while coping styles and personality traits influence rehabilitation success. Problem-focused coping helps to manage controllable stressors, and emotion-focused approaches support chronic condition management. Traits like extraversion, conscientiousness, and openness are linked to lower systemic inflammation and adherence to health guidelines [50,52]. Although these findings specifically address cardiac events, they may also apply to conditions like SCI. Both vascular events and SCI occur suddenly, and the episode can be traumatic. As a result, patients may develop a sedentary lifestyle, experience reduced psychological and physical motivation, and be inclined not to respect the therapeutic regimen [50]. Furthermore, individuals with severe medical conditions often exhibit reduced emotional expressiveness, such as alexithymia, which may impact their motivation for self-regulation and rehabilitation when dealing with illness or disability [21,23]. Specifically, patients with SCI may develop secondary alexithymic features due to the unconscious suppression or denial of their emotional experiences as a psychological response to their injury [24]. However, other research has found no significant increase in alexithymia scores in this population [25] and that alexithymia does not appear to be associated with the outcomes of inpatient therapy [26]. This suggests that the relationship between alexithymia and SCI may be more complex than initially assumed, requiring further investigation to determine how alexithymia influences rehabilitation outcomes in this population. These data highlight the dual role that psychological factors can play in either hindering or facilitating recovery, suggesting that interventions targeting mental health could improve overall rehabilitation outcomes. The implications of our findings are related to clinical practice. Previous studies have examined how psychological resources significantly impact the lives of individuals with SCI [53]. The challenges faced by individuals with SCI extend beyond the period of hospitalization. After returning home following institutional rehabilitation, individuals may experience heightened levels of depression due to increased dependency, limited social skills, inadequate social support beyond family, and restricted employment opportunities [17]. These findings underscore important considerations for the psychological management of individuals with SCI, highlighting the necessity of targeted psychological interventions during inpatient rehabilitation. Therefore, aligning rehabilitation strategies with individual psychological profiles can increase recovery efficacy. The integration of psychological assessments into routine rehabilitation protocols can lead to a more holistic approach to patient care. Addressing the emotional and psychological dimensions of recovery is essential for optimizing long-term outcomes. Mental health screening should become a standard component of rehabilitation assessments, allowing for the identification of patients at risk of lower outcomes due to psychological distress [54].

Clinically, this study underlines the necessity for a multidisciplinary approach to SCI rehabilitation, where mental health professionals work closely with physical therapists, neurologists, and rehabilitation specialists. Collaboration across disciplines would ensure that the psychological dimension is addressed concurrently with physical rehabilitation. It also highlights the need for long-term psychological support, as the effects of depression, anxiety, and other psychological factors may extend beyond the acute rehabilitation phase, impacting the long-term quality of life and functional independence of patients with SCI. Training rehabilitation staff in recognizing and addressing psychological factors can create a more supportive environment for patients, facilitating their engagement in the rehabilitation process.

The study presents some limitations. Firstly, the sample was recruited from a single specialized neurorehabilitation center, which may limit the generalizability of the results. The characteristics of patients in different contexts may vary significantly, affecting the applicability of the findings to other patient populations or less specialized settings. Therefore, future studies should be conducted in more diverse contexts to validate the findings and ensure that clinical recommendations can be effectively applied across a broader range of rehabilitation settings. Secondly, the complexity of the relationship between these factors and other aspects of the patient’s life may not have been fully explored. Considering the retrospective nature of the study, we analyzed data collected in our database that included the addressed variables. Future interesting studies could be aimed at investigating variables such as cognitive dimensions, social support, coping resources, or environmental variables in relation to the recovery process. In fact, the rehabilitation outcomes are related to the variables mentioned above [55]. Integrating such variables into future predictive models could enhance the understanding of the complexities of rehabilitation experiences and optimize intervention strategies. Future studies should aim to integrate these variables into predictive models, potentially increasing their accuracy and applicability across diverse populations. Moreover, the study’s cross-sectional design limits our ability to conclude changes over time and the causal relationships between psychological factors and rehabilitation outcomes. Longitudinal studies could provide deeper insights into how psychological factors evolve and impact recovery trajectories, thereby informing more targeted and effective rehabilitation approaches. Furthermore, the reliance on self-reported measures of psychological factors introduced potential biases related to response styles and social desirability. However, this is a retrospective analysis and in our clinical practice, we use the evaluation tools reported in the analysis. Future research should consider utilizing a combination of self-report measures and objective assessments to mitigate these biases and provide a more comprehensive understanding of the psychological dimensions of recovery after SCI, also including the above-mentioned variables not analyzed in this study. Addressing these limitations could enhance the clinical utility of this research. Finally, the predictive capacity of ANNs comes with challenges. As noted, ANNs often function as “black boxes,” making it difficult to discern the relationships between variables [13,32,56]. In particular, a weight was associated with each factor, and it represents the strength of the connection between this factor and the output, but the directionalities of these connections (if positive or negative) as well as the possible interactions among the factors were unclear and not clarified by the ANN. This is a disadvantage of using ANNs. However, the scientific literature already reports the results of multiple regressions and uses the values of beta coefficients to clarify the directions of the associations between input variables and the outcomes measured [13]. Another possible disadvantage is the fact that the results of ANNs are not uniquely determined given the input values and the output values for the training, potentially questioning their reliability. On the other hand, the capacity to predict the output is generally very high, as shown in our results. The cited limitations suggest the necessity for further explorations of alternative modeling techniques that might enhance interpretability while maintaining predictive power. Incorporating methods that provide clearer insights into the interactions among variables could inform better clinical decision-making and improve individualized patient management.

## 5. Conclusions

These results highlight the impact of psychological factors on rehabilitation outcomes following SCI through the application of ANNs. Our results suggest that in addition to established clinical predictors—such as the lesion level, the baseline SCIM score, and motor completeness—psychological factors significantly contribute to the prediction of the SCIM at discharge. Psychological variables accounted for 36.3% of the model’s predictive weight, with an enhanced contribution (40.9%) observed in traumatic SCI cases. Among the psychological variables, anxiety and depression emerged as dominant negative predictors, consistent with literature associating these factors with diminished recovery potential. Conversely, traits like self-esteem and emotional regulation functioned as protective factors, increasing functional outcomes and underlining the importance of psychological well-being in neurorehabilitation treatment. These findings advocate for the integration of psychological assessment tools into routine SCI rehabilitation protocols, aiming to tailor individualized therapeutic interventions that address both physical and mental health dimensions.

The high correlation coefficient between the predicted and actual SCIM scores at discharge, particularly in traumatic SCI cases, underscores the importance of psychological variables in refining prognostic accuracy. The model’s overall predictive strength highlights its utility in clinical practice, suggesting that psychological screening could enhance patient classification and optimize rehabilitation strategies. Future investigations should focus on expanding the model’s application to broader patient populations, validating these findings across different rehabilitation settings, and refining its predictive algorithms. These results underscore the relevance of a multidimensional approach to SCI management, integrating psychological parameters to enhance the accuracy of outcome prediction and the efficacy of rehabilitation interventions including psychological treatment.

## Figures and Tables

**Table 1:** Demographic, Clinical, and Psychological Variables and Their Weights from the Artificial Neural Network for Predicting SCIM at Discharge. SCIM: Spinal Cord Independence Measure. WISCI: Walking Index for Spinal Cord Injury. ASIA: American Spinal Injury Association.

Variables at Admission	All Patients		Patients with Traumatic Lesions	
Parameters	Descriptive Statistics	Weight ANN (n = 303)	Descriptive Statistics	Weight ANN (n = 140)
SCIM	21.8 ± 15.8	10.3%	17.5 ± 12.1	9.7%
WISCI	-	-	-	-
Age (years)	52.3 ± 18.2	8.0%	45.4 ± 18.8	10.0%
Lesion level	C: 33% T: 49% L: 18%	8.8%	C: 45% T: 42% L: 13%	6.2%
ASIA impairment scale	A: 31% B: 13% C: 27% D: 29%	10.7%	A: 40% B: 16% C: 21% D: 23%	9.6%
Motor completeness	42%	5.8%	53%	3.9%
Pressure sores	30.2%	3.8%	37.5%	5.3%
Complications	33.6%	4.0%	40.8%	2.0%
Deep vein thrombosis	-	-	-	-
Aetiology (traumatic)	44.6%	4.0%	100%	-
Pulmonary embolism	-	-	-	-
Having undergone surgical intervention	-	-	-	-
Hectopic ossification	1.5%	2.1%	1.3%	2.2%
Respiratory complications	2.4%	1.7%	2.0%	4.4%
Gender	M: 72% F: 28%	2.0%	M: 82% F: 18%	1.8%
Urological complications	0.6%	2.6%	0.7%	4.1%
Anxiety	−0.2 ± 1.0	9.5%	−0.2 ± 1.1	10.3%
Depression	0.3 ± 2.5	8.7%	0.3 ± 1.1	10.6%
Alexithymia	44.1 ± 11.6	7.7%	42.7 ± 10.1	7.9%
Self-esteem	32.6 ± 5.7	7.2%	33.0 ± 5.4	9.4%
Extroversion	1.6 ± 0.7	3.2%	1.7 ± 0.6	2.7%

## Data Availability

The data are available upon reasonable request to the corresponding author.
